# Variability of the chronic obstructive pulmonary disease key epidemiological data in Europe: systematic review

**DOI:** 10.1186/1741-7015-9-7

**Published:** 2011-01-18

**Authors:** Kokuvi Atsou, Christos Chouaid, Gilles Hejblum

**Affiliations:** 1 INSERM, U707, F-75012 Paris, France; 2 UPMC, Université de Paris 06, UMR S 707, F-75012 Paris, France; 3 AP-HP, Hôpital Saint Antoine, Service de Pneumologie, F-75012 Paris, France; 4 AP-HP, Hôpital Saint Antoine, Unité de Santé Publique, F-75012 Paris, France

## Abstract

**Background:**

Chronic obstructive pulmonary disease (COPD) is predicted to become a major cause of death worldwide. Studies on the variability in the estimates of key epidemiological parameters of COPD may contribute to better assessment of the burden of this disease and to helpful guidance for future research and public policies. In the present study, we examined differences in the main epidemiological characteristics of COPD derived from studies across countries of the European Union, focusing on prevalence, severity, frequency of exacerbations and mortality, as well as on differences between the studies' methods.

**Methods:**

This systematic review was based on a search for the relevant literature in the Science Citation Index database via the Web of Science and on COPD mortality rates issued from national statistics. Analysis was finally based on 65 articles and Eurostat COPD mortality data for 21 European countries.

**Results:**

Epidemiological characteristics of COPD varied widely from country to country. For example, prevalence estimates ranged between 2.1% and 26.1%, depending on the country, the age group and the methods used. Likewise, COPD mortality rates ranged from 7.2 to 36.1 per 10^5 ^inhabitants. The methods used to estimate these epidemiological parameters were highly variable in terms of the definition of COPD, severity scales, methods of investigation and target populations. Nevertheless, to a large extent, several recent international guidelines or research initiatives, such as GOLD, BOLD or PLATINO, have boosted a substantial standardization of methodology in data collection and have resulted in the availability of more comparable epidemiological estimates across countries. On the basis of such standardization, severity estimates as well as prevalence estimates present much less variation across countries. The contribution of these recent guidelines and initiatives is outlined, as are the problems remaining in arriving at more accurate COPD epidemiological estimates across European countries.

**Conclusions:**

The accuracy of COPD epidemiological parameters is important for guiding decision making with regard to preventive measures, interventions and patient management in various health care systems. Therefore, the recent initiatives for standardizing data collection should be enhanced to result in COPD epidemiological estimates of improved quality. Moreover, establishing international guidelines for reporting research on COPD may also constitute a major contribution.

## Background

Chronic obstructive pulmonary disease (COPD) is a preventable disease characterized by partially irreversible airflow limitation due to progressive inflammation of the lower airways. Smoking is the main risk factor [1,2]. COPD is a common disease whose age-standardized mortality rate increased during the 1990s, and it is predicted to be a major cause of death worldwide during the next two decades [3-5]. With such a high burden on the healthcare system, emphasis on better diagnosis and management of the disease must be achieved and reliable epidemiological data on the prevalence and severity of COPD and its exacerbations are crucial to guide decisions. Indeed, international comparisons of COPD prevalence, mortality rates and management of acute exacerbations may help to identify the most effective healthcare systems in this setting.

Differences in healthcare systems and criteria make it difficult to compare data across different countries [6,7]. Efforts have been made to standardize prevalence estimates through the international Latin American Project for the Investigation of Obstructive Lung Disease (PLATINO) and Burden of Obstructive Lung Disease (BOLD) study initiatives, for example [8,9]. To obtain a representative picture of the epidemiological situation in Europe and what has been achieved so far, we reviewed the literature on COPD in Europe, focusing on prevalence, severity, exacerbations and mortality. The goal of the study was to examine the differences in these epidemiological estimates across countries in light of the differences in methods used from one study to another. Therefore, we collected the following features associated with the epidemiological estimates reported: publication date, type of population, sample size and definitions used (for example, severity scale used, exacerbation definition used). This review strongly suggests that establishing reporting guidelines dedicated to COPD studies may constitute a major contribution to enhancing the value of COPD research and management by facilitating comparisons between studies.

## Methods

### Document search

We sought documents on the prevalence, mortality, severity and exacerbations of COPD in the Science Citation Index database via the Web of Science (WOS) (Thomson Reuters, New York, NY, USA). The search was limited to documents published between 1991 and 2009, focusing on the epidemiology of COPD (prevalence, severity, exacerbations and mortality) in different populations (general population, patients followed up by a general practitioner and/or chest specialist, ambulatory patients and inpatients). We also extracted data from the European statistical database (Eurostat) [10].

WOS was queried using a four-step strategy as follows:

1. TS = COPD or chronic obstructive pulmonary disease or chronic bronchitis

2. TS = Prevalence or severity or mortality or exacerbation

3. TI = Cancer

4. (2 AND 1) NOT 3

where TI is a term search limited to the title of the document and TS is a term search in the title, abstract and keywords.

### Document triage and selection

We applied the following exclusion criteria to the WOS documents obtained using the above strategy: We excluded documents concerning non-European Union countries, documents that were not original articles or reviews and documents with WOS subject categories not relevant to this study (for example, biophysics, biochemistry, microbiology, psychology, social issues, bioinformatics, toxicology). Finally, document eligibility was completed by deeper examination of the remaining articles. Articles not dealing primarily with the epidemiology of COPD (that is, prevalence, severity, mortality, exacerbations) or not specifying sufficiently important definitions related to the epidemiological estimates (for example, diagnostic tests used and target population, including inclusion and exclusion criteria) were excluded from the review. A comparable search in the MEDLINE database via the PubMed interface did not retrieve any additional documents.

The Eurostat database was used to extract age-standardized COPD mortality rates (deaths per 100,000 inhabitants) for 2007 by using code J40-47 (death as a result of lower respiratory tract disease) and by excluding codes J45 and J46 (death as a result of asthma). Death as a result of bronchiectasis (J47) was not excluded, because the corresponding number of cases was considered minor.

### Analysis of the collected information

Considering each of the reported epidemiological estimates (prevalence, severity, exacerbations and mortality), we collected several associated features: publication date, type of population under study, sample size and definitions used (for example, severity scale used, exacerbation definition used). We then listed each estimate report with its associated feature described above to obtain a picture of the variability of estimates across countries in light of the methodological differences between studies.

## Results

### Information included in the review

**Figure 1:**
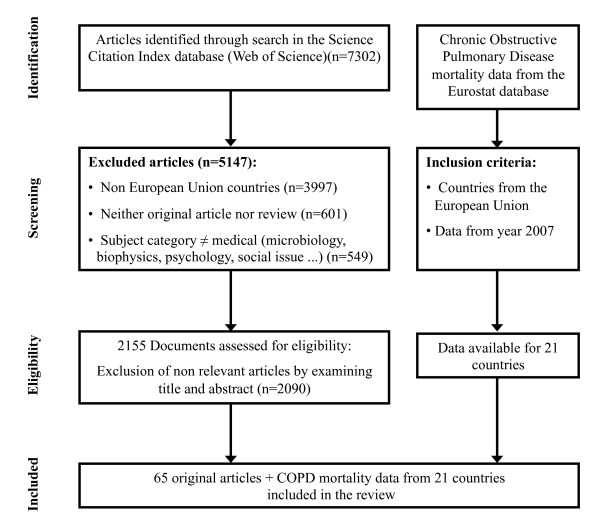
**Flowchart of the process for collecting information included in the review**.

The process for including final information used in the review is detailed in Figure 1. The initial literature search resulted in 7,302 WOS documents, and after applying the exclusion criteria and eligibility steps, 65 articles (61 in English, 3 in Spanish [11-13] and 1 in French [14]) were finally included in the study (Figure 1). COPD mortality data in the Eurostat database were available for 21 countries of the European Union.

### Prevalence

COPD prevalence data can be categorized into four types according to the criteria used to define the disease (Table 1; see also a graph of these data in Additional file 1, Figure A1), namely, symptoms [15-17], physician reports [18-23], spirometry [8,11-13,24-39] and models [40-42].

**Table 1:** COPD prevalence data^a^

Criteria used to define COPD	Country (sample size)	Study population	**Age**^**b **^**(yr)**	Prevalence (%)
Symptoms^c^				
Cerveri *et al.*, 2003 [15]	Italy (18,645)	General population	20-44	9.5
Huchon *et al.*, 2002 [16]	France (14,076)	General population	≥25	4.1
Cerveri *et al.*, 2001 [17]	16 countries (14,819)	General population	20-44	2.6
				
Physician reports				
Cazzola *et al.*, 2009 [18]	Italy (15,229)	General population	NS	2.5
Schirnhofer *et al.*, 2007 [19]	Austria (1,258)	General population	≥40	5.6
Montnemery *et al.*, 2006 [20]	Sweden (3,692)	General population	20-59	3.6
Hedman *et al.*, 1999 [21]	Finland (3,102)	General population	18-65	3.7
Montnemery *et al.*, 1998 [22]	Sweden (8,469)	General population	20-59	4.6
Lundback *et al.*, 1991 [23]	Sweden (6,610)	General population	35-66	4.1
				
Functional respiratory tests				
Miravitlles *et al.*, 2009 [24]	Spain (4,274)	General population	56.6 (10.7)	10.2
Van Durme *et al.*, 2009 [25]	Netherlands (7,983)	General population	≥55	11.6
Hansen *et al.*, 2008 [26]	Denmark (4,757)	General population	45-84	12.0
Bednarek *et al.*, 2008 [27]	Poland (1,960)	Primary care	56.7 (11.6)	9.3
Roche *et al.*, 2008 [28]	France (4,764)	Health prevention center	59.9 (10.1)	2.6
Buist *et al.*, 2007 [8]	Austria (1,258)	General population	≥40	26.1^d^
	Germany (683)			13.3
	Poland (526)			22.1
	Norway (638)			18.8
Shahab *et al.*, 2006 [29]	United Kingdom (8,215)	General population	55.5 (13.5)	13.3
Stavem *et al.*, 2006 [30]	Norway (1,619)	Occupational cohort	49.8 (5.5)	16.4
Sichletidis *et al.*, 2005 [31]	Greece (6,112)	General population	21-80	5.6
Murtagh *et al.*, 2005 [32]	Ireland (2,484)	General population	53.3 (8.6)	6.3
Tzanakis *et al.*, 2004 [33]	Greece (888)	General population	≥35	8.4
Hasselgren *et al.*, 2001 [34]	Sweden (4,814)	General population	43 (14.8)	2.1
Peña *et al.*, 2000 [35]	Spain (3,978)	General population	40-69	9.1
Viegi *et al.*, 2000 [36]	Italy (1,727)	General population	≥25	11.0 or 18.3
Jaen *et al.*, 1999 [11]	Spain (497)	General population	20-70	7.2
Dickinson *et al.*, 1999 [37]	United Kingdom (353)	General population	68.25	9.9
Marco Jordán *et al.*, 1998 [12]	Spain (460)	General population	40-60	6.8
Renwick & Connolly, 1996 [38]	United Kingdom (783)	General practitioner	66.1	9.0
Brotons *et al.*, 1994 [13]	Spain (642)	General population	35-65	6.4
Bakke *et al.*, 1991 [39]	Norway (1,275)	General population	42 (16.1)	4.5
				
Models				
Peabody *et al.*, 2005 [40]	Spain	Total population	≥30	6.2
	Norway			6.3
	Poland			6.7
Feenstra *et al.*, 2001 [41]	Netherlands	Total population	≥20	1.5
Stang *et al.*, 2000 [42]	Spain	Total population	≥45	10.3
	Italy			11.1
	France			10.4
	United Kingdom			15.0

^a^Prevalence estimates are based on symptoms (cough and sputum at least 3 months each year), physician reports, functional respiratory tests (FEV_1_/FVC ratio <70%, FEV_1 _<80% of predicted, FEV_1_/FVC ratio <65%, FEV_1_/FVC ratio <70% or FEV_1_/FVC ratio <88% (male)/89% (female)) and models (general population). ^b^Age was reported in various ways distinguished here as follows: "≥ x", minimum age; "x" or "x (y)", mean age or mean age (SD); "x-y", age range i.e. min-max. ^c^These patients had chronic bronchitis. ^d^These data are also reported in Schirnhofer *et al.*, 2007 [19]. Abbreviations used: COPD, chronic obstructive pulmonary disease; NS, not specified; FEV_1_, maximum expiratory volume in 1 second; FVC, forced vital capacity.

Surveys based on symptoms (cough and expectoration on most days for as much as three months per year and for at least two successive years) were used to estimate the prevalence of chronic bronchitis (initial stage of COPD). This prevalence ranged from 2.6% to 9.5% in adults under 44 years of age [15-17]. Major variability was noted across European countries, with prevalence rates ranging from 0.7% to 9.7% (median, 2.6%) in a study covering 16 countries [17]. There were also significant regional differences within a given country [17]. The prevalence of chronic bronchitis is reported to be significantly lower among women than among men in all countries (2.8% versus 3.7%; *P *< 0.001) [17]. The prevalence increased gradually from nonsmokers to "moderate-heavy" smokers (≥15 packs-yrs) and from higher socioeconomic classes to the unemployed and blue-collar workers [15-17].

Studies [18-23] based on diagnoses of chronic bronchitis by healthcare professionals have produced less varying results, with prevalence rates ranging from 3.7% to 5.6% among adults. In studies based on respiratory function, such as the ratio of the maximum expiratory volume in 1 second (FEV_1_) over forced vital capacity [8,11-13,24-39] or the difference between measured and predicted FEV_1 _[37], the prevalence ranged from 2.1% [37] to 26.1% [8,19], but this large range may be attributed in part to the different types of population in which the prevalence was estimated. However, differences in population characteristics only partly explain the variability of the reported prevalence estimates. Even if one limits the analysis to patients over 40 years of age, the prevalence of the COPD still varies widely, from 4.5% in Norway [39] to 26.1% in Austria [8]. The functional criteria used to diagnose COPD also vary from one study to another, depending on the classification used: American Thoracic Society (ATS) [43], British Thoracic Society (BTS) [44], European Respiratory Society Task Force (ERS) [45], or Global Initiative for Chronic Obstructive Lung Diseases (GOLD) [46]. In the international BOLD study [8], based on the general population and on the current GOLD guidelines, the prevalence estimates issued from the 12 countries under study significantly varied from one site to another, with differences between men and women likely to reflect gender smoking rates. Considering the sites from the five European countries participating in the BOLD study, prevalence estimates ranged from 13.3% in Germany to 26.1% in Austria. In all, six of the nine papers that used the definition of COPD proposed by the GOLD initiative refer to the general population [8,19,24-26,29], and with respect to these data, the prevalence estimates range between 10.2% in Spain [24] and 26.1% in Austria [8].

Several authors have proposed model-based approaches for estimating COPD prevalence [40-42]. These models combine demographic data with smoking rates [40-42], data on respiratory function in the general population [40,42] and other risk factors such as air pollution and low socioeconomic status [40]. In these studies, the prevalence varied from 1.5% to 15% in the general population, and a given model [42] applied to the population over 45 years of age in four European countries resulted in estimates varying from 10.3% in Spain to 15% in the United Kingdom.

### Severity

**Figure 2:**
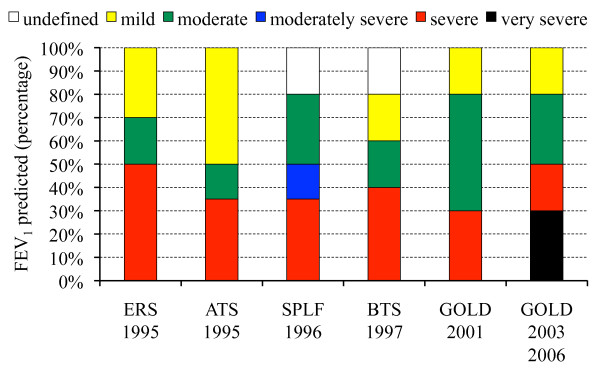
**The most popular chronic obstructive pulmonary disease classifications: ERS, European Respiratory Society; ATS, American Thoracic Society; SPLF, Société de Pneumologie de Langue Française; BTS, British Thoracic Society; GOLD, Global Initiative for Chronic Obstructive Lung Disease; FEV_1_, forced expiratory volume in 1 second**. ATS, BTS, ERS and SPLF classifications define three stages of severity between 0% and 100% (ATS and ERS) or 80% (BTS and SPLF) of predicted FEV_1_. The most recent classification is the GOLD classification, which was initially proposed in 2001 and has been modified twice, in 2003 and 2006. In 2003, the GOLD classification evolved from three to four stages plus a supplementary stage 0 defining patients at risk (that is, the presence of chronic cough and sputum, but no obstruction together with FEV_1 _over forced vital capacity ratio >70%; stage 0 is not shown in the figure), and stage 0 was not kept in the GOLD classification system in 2006.

The reported epidemiology of COPD severity [8,14,27,29,33,36,47-57] depends on whether the analysis concerns the general population [8,29,33,36,47-50], patient follow-up by a general practitioner and/or chest specialist [14,27,51-54] or inpatients (Table 2; see also a graph of these data in Additional file 2, Figure A2) [55-57]. Moreover, results were also reported according to various severity scales (Figure 2).

**Table 2:** Severity of COPD^a^

				**Severity**^**c **^**(%)**			
				
Study population	Country (sample size)	Criteria	**Age**^**b **^**(yr)**	Stage 1	Stage 2	Stage 3	Stage 4
General population							
						
Buist *et al.*, 2007 [8]	Austria (1,349)	GOLD 2006	≥40	59.4	35.2	5.4	
	Germany (713)			55.3	38.6	6.1	
	Poland (603)			50.7	40.7	8.6	
	Norway (707)			55.8	37.8	6.4	
Shahab *et al.*, 2006 [29]	United Kingdom (8,215)	GOLD 2006	55.5 (13.5)	41.6	43.9	14.5	
						
Lindberg *et al.*, 2006 [47]	Sweden (1,237)	GOLD 2006	46-77	57.0	37.0	5.0	1.0
		BTS 1997		65.0	27.0	8.0	NA
						
De Marco *et al.*, 2004 [48]^d^	Belgium (1,122)	GOLD 2003	20-44	58.6	41.4		ND
	Denmark (394)			56.4	43.6		ND
	France (2,137)			66.7	33.3		ND
	Germany (1,983)			64.8	35.2		ND
	Italy (910)			69.4	30.6		ND
	Ireland (454)			75.3	24.7		ND
	Netherlands (1,362)			74.3	25.7		ND
	Norway (969)			40.7	59.3		ND
	Spain (1,942)			59.2	40.8		ND
	Sweden (1,859)			79.3	20.7		ND
	Switzerland (853)			76.3	23.7		ND
	United Kingdom (1,198)			69.8	30.2		ND
						
Tzanakis *et al.*, 2004 [33]	Greece (888)	ERS 1995	≥35	58.2	25.6	16.2	NA
Jansson *et al.*, 2002 [49]	Sweden (212)	BTS^e^	28-29	75.0	25.0	0	0
			43-44	46.6	46.6	6.8	0
			49-50	16.0	64.0	12.0	8.0
			58-59	15.4	57.7	15.4	11.5
			64-65	6.9	29.3	41.4	22.4
			73-74	14.3	21.4	47.6	16.7
			79-80	9.8	34.2	48.8	7.3
Viegi *et al.*, 2000 [36]	Italy (1,727)	ERS 1995	≥25	81.0	14.0	5.0	NA
		ATS 1995		98.2	1.8	0	NA
					
		Clinical^f^		86.1	13.9		
					
Von Hertzen *et al.*, 2000 [50]^g^	Finland (7,217)		≥30	59.8	34.1	6.1	NA

General practitioner and/or chest specialist							
Izquierdo *et al.*, 2009 [51]	Spain (3,619)	GOLD 2006	67.0 (10.8)	20.1	54.0	22.1	3.8
Bednarek *et al.*, 2008 [27]	Poland (1,960)	GOLD 2006	56.7 (11.6)	30.6	51.4	15.3	2.7
Hoogendoorn *et al.*, 2006 [52]	Sweden (481)	GOLD 2006	65.5	30.0	51.0	17.0	2.0
Piperno *et al.*, 2005 [14]	France (3,411)	SPLF 1996	58.4 (9.9)	31.3	50.2	18.5	NA
Detournay *et al.*, 2004 [53]	France (255)	SPLF 1996	67.1	56.0	27.0	17.0	NA
Soriano *et al.*, 2000 [54]	United Kingdom (23,277)	Prescriptions	66.7 (15.5)	35.5	56.4	8.1	NA
							
Hospital							
Soler-Cataluña *et al.*, 2005 [55]	Netherlands (304)	GOLD 2006	71 (9)	6.6	35.8	33.6	24.0
Tsoumakidou *et al.*, 2004 [56]	Greece (67)	ERS 1995	69.0	0	4.5	95.5	NA
		BTS 1997	69.5	1.5	14.9	83.6	NA
		ATS 1995	67.6	4.5	28.3	67.2	NA
						
		GOLD 2001	69.5	0	58.2		41.8
Kornmann *et al.*, 2003 [57]	Germany (1,434)	GOLD 2001	{55}	7.9	73.0		19.1

^a^COPD severity data are reported for the general population, inpatients, general practitioner reports, general practitioner and chest specialist reports or are taken from medical databases. ^b^Age was reported in various ways distinguished here as follows: "≥ x", minimum age; "x" or "x (y)", mean age or mean age (SD); "x-y", age range i.e. min-max; "{x}", median age. mean age (±SD); median age; age range, min-max. ^c^ERS 1995, ATS 1995, BTS 1995 and SPLF 1995 classifications define only three stages. ^d^In this study, based on the GOLD 2003 severity scale, the estimates for stage 0 defining patients at risk of COPD ranged from 51.7% in Switzerland to 89.8% in Spain. ^e^Patients with both FEV_1_/FVC <70% and FEV_1 _≥80% (considered as having COPD in the GOLD 2006 classification but not in the BTS 1995 classification) were also included in the study. ^f^Airway obstruction of any degree was defined by FEV_1_/FVC ratio <70% and the severity of obstruction was graded according to the FEV_1 _value: mild (FEV_1 _≥70% of predicted) and moderate to severe (FEV_1 _<70% of predicted). ^g^Airway obstruction of any degree was defined by FEV_1_/FVC ratio <80% and the severity of obstruction was graded according to the FEV_1_/FVC value: 70-79%, minimal to mild; 50-69%, moderate; <50%, severe. Abbreviations used: COPD, chronic obstructive pulmonary disease; ERS 1995, European Respiratory Society 1995 classification; ATS 1995, American Thoracic Society 1995 classification; BTS 1995, British Thoracic Society 1995 classification; SPLF 1995, Société de Pneumonologie de Langue Française 1995 classification; GOLD 2001, GOLD 2003 and GOLD 2006, Global Obstructive Lung Disease 2001, 2003 and 2006 classifications; ND, not defined; NA, not applicable; FEV_1_, maximum expiratory volume in 1 second; FVC, forced vital capacity.

In general patient populations, early-stage disease predominates among people over 40 years of age, with rates of GOLD 2006 stage 1 disease (FEV_1 _≥80% of predicted) and stage 2 disease (80% < FEV_1 _≤ 50% of predicted), respectively, ranging from 50.7% and 40.7% in Poland to 59.4% and 35.2% in Austria [8]. In a study based on the GOLD 2003 severity scale and on a younger population (ages 20 to 44 years), estimates substantially varied from one country to another: from 51.7% in Switzerland to 89.8% in Spain for stage 0 and from 3.4% in Italy to 16.8% in Denmark for stages 2 and 3. The authors had no clear explanation for these differences [48]. The proportion of advanced stages increases with age, peaking at age 60 years. Thus, in a Swedish study based on the BTS classification [49], the percentage of stage 4 disease, which was negligible below age 40 years, reached 22.4% at age 58 or 59 years before decreasing again to 7.3% at 73 or 74 years of age. This increase with age is observed in both men and women [8]. The relationship between tobacco consumption and GOLD stage 2 disease or higher was less clear, partly owing to interference by age [8]. Moderate forms of the disease are more frequent in cohorts of patients followed by general practitioners or specialists, with observed frequencies ranging from 27% to 56.4% [14,27,51-54].

Analyses of severity among COPD inpatients show, quite logically, more frequent advanced forms of the disease, with a dramatic decrease in the proportion of patients with mild COPD (reported proportions ranging from 0% to 7.9%). The existence of several severity scales leads to major variations in the reported epidemiology of COPD severity, even when considering a given type of population. Depending on whether the ERS, ATS, BTS or GOLD criteria are used (Table 2), the percentage of early stages in the general population is, respectively, 80%, 98%, 65% and 55%, while advanced stages represent, respectively, 95%, 67%, 85% and 58% of cases among COPD inpatients [36,47,56].

In general, there is a relatively low variability in the severity estimates based on GOLD criteria and derived from a given type of population. For example, when considering the general population of patients who are at least 40 years old, the severity distributions of patients in Norway [8], Germany [8] and Sweden [47] are similar. In the same way, the severity distributions in patients recruited from general practitioners and/or specialists in Sweden [52] and Poland [27] are comparable.

### Exacerbations

Tables 3 and 4 list the 22 reports mentioning the frequency of COPD exacerbations in European patients according to severity stage [53,55,58-77]. The definition of exacerbation substantially varied from one study to another (Table 3). The mean number of exacerbations per person and per year ranges from 0.6 to 3.5 [63,72] according to the disease stage and the age group (Table 4). Borg *et al.*'s model [77], in which parametric values were derived from the literature, used mean frequencies of exacerbations of, respectively, 0.05 and 1.47 per person per year at GOLD stages 1 and 4. According to Soler-Cataluña *et al.*[55], 26% of very severely affected patients have at least three exacerbations per year compared to 0% of mildly affected patients. The number of exacerbations can be reduced by treatment [60,66]. Exacerbations are usually categorized as mild (symptoms treated at home or easily tolerated), moderate (treatment in a hospital or affecting daily activity) or severe (specialist treatment or inability to work). More severe disease stages are associated with more severe exacerbations [60,66]. In Borg *et al.*'s model [77], the average frequencies of severe exacerbations are 0.01 and 0.33 per patient per year at GOLD stage 1 and GOLD stage 4, respectively. Such estimates have been confirmed by another observational study [55].

**Table 3:** Exacerbation definitions^a^

Diagnostic method	Description of symptoms
Treatment	
Burge *et al.*, 2003 [58]^b^	Chest problem requiring treatment with oral corticosteroids and/or antibiotics as defined by the treating physician
Calverley *et al.*, 2003 [59]	Worsening of COPD symptoms that required treatment with antibiotics, oral corticosteroids or both
Jones *et al.*, 2003 [60]^b^	Chest problems requiring treatment with antibiotics and/or oral corticosteroids
Andersson *et al.*, 2002 [61]	Increased dose of current treatment and/or treatment with antibiotics or systemic corticosteroids and/or general practitioner or outpatient visit or hospital admission
Burge *et al.*, 2000 [62]^b^	Worsening of respiratory symptoms that required treatment with oral corticosteroids, antibiotics or both as judged by the general practitioner
Symptoms	
Effing *et al.*, 2009 [63]	Presence for at least 2 consecutive days of an increase in any two major symptoms or an increase in one major and one minor symptom
Worth *et al.*, 2009 [64]	A complex of at least two respiratory adverse events with a duration of more than 3 days
Schermer *et al.*, 2009 [65]	Episode with one or more subsequent unscheduled contacts with either a general practitioner or a chest physician because of worsening of respiratory symptoms
O'Reilly *et al.*, 2006 [66]^c^	Symptom-based: Symptom score of at least 2 for 2 consecutive days with no score for at least two of these symptoms in the previous 5 days Healthcare-based: Need to take antibiotics and/or oral corticosteroids for chest problem
Wilkinson *et al.*, 2006 [67]	Not defined but specified as symptom-based
Donaldson *et al.*, 2003 [68]	An increase in either two or more major symptoms or any one major symptom plus any minor symptoms occurring on 2 or more consecutive days
Seemungal *et al.*, 2000 [69]	Presence for at least 2 consecutive days of an increase in any two major symptoms or an increase in one major and one minor symptom
Treatment and symptoms	
Seemungal *et al.*, 2008 [70]	Sustained worsening of baseline respiratory symptoms for at least 2 days that required treatment with oral corticosteroids and/or antibiotics
Tashkin *et al.*, 2008 [71]	Increase or new onset of more than one respiratory symptom (cough, sputum, sputum purulence, wheezing or dyspnea) lasting 3 days or more and requiring treatment with an antibiotic or a systemic corticosteroid
Calverley *et al.*, 2008 [72]	Clinically significant worsening of COPD symptoms requiring treatment with antibiotics and/or systemic steroids
Wedzicha *et al.*, 2008 [73]	Symptom worsening that required treatment with oral corticosteroids and/or antibiotics or required hospitalization
Dusser *et al.*, 2006 [74]	Onset of at least one clinical descriptor (worsening of dyspnea, cough or sputum production, appearance of purulent sputum, fever (>38°C), appearance of a new chest radiograph abnormality) lasting ≥2 days and requiring a new prescription or an increase in the dose of β_2_-agonists, antibiotics, corticosteroids or bronchodilators
Soler-Cataluña *et al.*, 2005 [55]	Sustained increase in respiratory symptomatology compared with baseline requiring modification of regular medication and hospital treatment (acute exacerbation of COPD)
Oostenbrink *et al.*, 2004 [75]	Complex of respiratory symptoms (new onset or worsening of more than one symptom such as cough, sputum, dyspnea or wheeze) lasting for ≥3 days
Brusasco *et al.*, 2003 [76]	Complex of respiratory symptoms (new onset or an increase in at least one of the following: cough, sputum, dyspnea, wheeze, chest discomfort) lasting at least 3 days and usually associated with therapeutic intervention
Model	
Borg *et al.*, 2004 [77]	Increase in any two major symptoms (dyspnea, sputum purulence, sputum amount) or an increase in one major and one minor symptom (wheeze, sore throat, cough, and symptoms of a common cold, which were nasal congestion and/or discharge) for at least 2 consecutive days
Not defined	
Detournay *et al.*, 2004 [53]	ND

^a^Criteria used to define exacerbations were symptoms, treatment, symptoms and treatment or model-based. COPD stages are defined according to GOLD [53,55,59-62,66-69,74-77,90], BTS [61] and SPLF [53] criteria. ^b^These three articles concern the same patients (the Inhaled Steroids in Obstructive Lung Disease trial). ^c^Healthcare-based definition of exacerbation corresponds to the "treatment and symptoms"-based definition. Abbreviations used: COPD, chronic obstructive pulmonary disease; BTS, British Thoracic Society classification; GOLD, Global Obstructive Lung Disease classification; SPLF, Société de Pneumonologie de Langue Française classification.

**Table 4:** COPD exacerbations^a^

Exacerbation definition group **(study type**^**b**^**)**	Country (sample size)	Classification (stage of COPD)	Mean number of exacerbations per patient **and per year**^**c**^	Treatment
Treatment				
Burge *et al.*, 2003 [58]^d ^(CT)	United Kingdom (524)	GOLD (2 or 3)	1.6-1.7	Placebo
			1.1-1.4	Fluticasone propionate
Calverley *et al.*, 2003 [59] (CT)	25 countries (1,974)	GOLD (2 or 3)	1.3	Placebo
			1.0	Salmeterol
			1.0	Fluticasone
			1.0	Salmeterol and fluticasone
Jones *et al.*, 2003 [60]^d ^(CT)	United Kingdom (751)	GOLD (1 or 2)	1.0	Placebo
			0.7	Fluticasone
		GOLD (3 or 4)	1.7	Placebo
			1.5	Fluticasone
Andersson *et al.*, 2002 [61] (CT)	Sweden (191)	GOLD/BTS	1.2	
Burge *et al.*, 2000 [62]^d ^(CT)	United Kingdom (751)	GOLD (2 or 3)	1.9 (2.6)	Placebo
			1.4 (1.9)	Fluticasone
Symptoms				
Effing *et al.*, 2009 [63] (CT)	Netherlands (142)	GOLD (2 or 3)	3.5 (2.7)	
Worth *et al.*, 2009 [64]^e ^(CT)	Germany (220)	GOLD (3 or 4)	0.9	Placebo
			0.4	Cineole
Schermer *et al.*, 2009 [65] (CT)	Netherlands (286)	GOLD (1-3)	0.7	Placebo
			0.9	Fluticasone
			1.0	*N*-acetylcysteine
				
O'Reilly *et al.*, 2006 [66] (OS)	United Kingdom (309)	GOLD (1 or 2)	2.2 [1.9-2.7]	Symptom-defined
			2.3 [2.0-2.8]	Healthcare-defined
		GOLD (3 or 4)	2.5 [2.1-2.9]	Symptom-defined
			3.2 [2.8-3.7]	Healthcare-defined
Wilkinson *et al.*, 2006 [67] (OS)	United Kingdom (74)	GOLD (2 or 3)	2.5 {1.3-3.8}	
Donaldson *et al.*, 2003 [68] (OS)	United Kingdom (132)	GOLD (2 or 4)	2.5 {1.3-3.9}	
Seemungal *et al.*, 2000 [69] (OS)	United Kingdom (101)	GOLD (2 or 4)	2.4 {1.3-3.8}	
				
Treatment and symptoms				
Seemungal *et al.*, 2008 [70] (CT)	United Kingdom (109)	GOLD (2 or 3)	2.0	Placebo
			1.0	Erythromycin
Tashkin *et al.*, 2008 [71] (CT)	37 countries (5,993)	GOLD (2-4)	0.8	Placebo
			0.7	Tiotropium
Calverley *et al.*, 2008 [72] (CT)	11 countries (911)	GOLD (2 or 3)	1.0	Placebo
			0.6	Mometasone furoate
Wedzicha *et al.*, 2008 [73] (CT)	20 countries (1,323)	GOLD (3 or 4)	1.3	SFC
			1.3	Tiotropium
Dusser *et al.*, 2006 [74] (CT)	France (1,010)	GOLD (1 or 2)	2.0	Placebo
			1.2	Tiotropium
		GOLD (3 or 4)	1.8	Placebo
			2.7	Tiotropium
Soler-Cataluña *et al.*, 2005 [55] (OS)	Spain (304)	GOLD (1)	(75, 25, 00)^f^	
		GOLD (2)	(60, 35, 05)^f^	
		GOLD (3)	(56, 32, 12)^f^	
		GOLD (4)	(34, 40, 26)^f^	
Oostenbrink *et al.*, 2004 [75] (CT)	Netherlands and Belgium (519)	GOLD (1-4)	1.0 (0.1)	Placebo
			0.7 (0.1)	Fluticasone
Brusasco *et al.*, 2003 [76] (CT)	18 countries (1,207)	GOLD (2 or 3)	1.5	Placebo
			1.2	Salmeterol
			1.1	Tiotropium
				
Model				
Borg *et al.*, 2004 [77] (M)	Netherlands	GOLD (1)	(0.05, 0.07, 0.01)^g^	
		GOLD (2)	(1.01, 1.31, 0.14)^g^	
		GOLD (3)	(1.06, 1.45, 0.17)^g^	
		GOLD (4)	(1.47, 1.72, 0.33)^g^	
				
Not defined				
Detournay *et al.*, 2004 [53] (OS)	France (255)	Moderate	1.7	
		Moderate to	1.5	
		severe		
		Severe	2.0	

^a^Criteria used to define exacerbations were symptoms, treatment, symptoms and treatment or model-based. COPD stages are defined according to GOLD [53,55,59-62,66-69,74-77,90], BTS [61] and SPLF [53] criteria. ^b^Study type reported as CT, clinical trial; OS, observational study; M, model. ^c^Mean numbers of exacerbations per person and per year were reported in various ways: mean exacerbation or mean exacerbation (±SD) or mean exacerbation {min-max} or [95% confidence interval]. ^d^These three articles concern the same patients (the Inhaled Steroids in Obstructive Lung Disease trial). ^e^These estimates are based on 6 months of follow-up. The mean exacerbation in both groups during the previous year was 3.2. ^f^This article does not give the mean number of exacerbations but the proportion of patients who had, respectively, no acute exacerbations, one, two or three or more. ^g^Mean number of exacerbations per person and per year reported separately for mild, moderate and severe exacerbations. Abbreviations used: COPD, chronic obstructive pulmonary disease; BTS, British Thoracic Society classification; GOLD, Global Obstructive Lung Disease classification; SPLF, Société de Pneumonologie de Langue Française classification; SFC, salmeterol and fluticasone propionate (anti-inflammatory drug combination); ISOLDE, the Inhaled Steroids in Obstructive Lung Disease trial.

### Mortality

As shown in Table 5 (see also a graph of these data in Additional file 3, Figure A3), reported estimates of COPD mortality in the general population vary from one country to another, but data interpretation must be done carefully. First, the International Classification of Diseases (ICD) has evolved with time, and the underlying classification or codes used for estimating mortality (for example, Hurd *et al.*[78] included codes 495 and 496 corresponding to asthma) are never similar from one study to another. Overall, mortality estimates have increased with time. However, increases in COPD mortality and features related to coding of mortality may both be involved in this increase, with relative parts hardly quantifiable. At present, the fairest comparison between countries should be made with 2007 European COPD-related mortality estimates issued by Eurostat [10]. The estimates ranged from 7.2 per 100,000 inhabitants in France to 36.1 per 100,000 inhabitants in Hungary, and mortality was between 1.3 (Sweden) and 13 (Malta) times higher in men than in women. Mortality estimates among hospital inpatients are high, as expected, at around 25% [79,80] or even higher in patients with comorbidities [81]. The mortality of hospitalized patients with COPD increases with the number of exacerbations: In the study by Soler-Cataluña *et al.*[55], patients with 0, 1 or 2 and 3 or more acute COPD exacerbations per year had 36-month survival probabilities of 0.86, 0.70 and 0.42, respectively.

**Table 5:** COPD age-standardized mortality rates^a^

				Annual mortality rate (per 100,000 inhabitants)			
					
Population	Country	Year(s)	Age (yr)	Global	Male	Female	Classification codes used
General population							
Eurostat^b,c ^[10]	Austria	2007		19.4	30.8	12.1	ICD 10 (J40-J44, J47)
	Bulgaria			15.8	26.7	07.9	
	Cyprus			09.3	15.5	04.7	
	Czech Republic			14.7	24.1	08.5	
	Estonia			10.7	24.2	04.4	
	Finland			12.9	25.3	05.4	
	France			07.2	12.5	03.8	
	Germany			16.3	24.9	10.8	
	Greece^d^			10.9	14.8	07.9	
	Hungary			36.1	56.5	23.6	
	Lithuania			22.4	49.0	08.5	
	Latvia			09.7	22.4	03.0	
	Malta			16.0	35.4	02.7	
	Netherlands			26.0	39.6	18.7	
	Poland			16.8	32.0	07.8	
	Romania			20.6	33.8	11.4	
	Spain			18.4	37.1	06.1	
	Sweden			15.1	17.6	13.6	
	Slovakia			12.8	24.5	05.9	
	Slovenia			13.2	23.4	07.5	
	United Kingdom			28.4	35.5	23.7	
Hurd *et al.*, 2000 [78]	Austria	1997	35-74		32	6	ICD 9 (490-496)
	Bulgaria	1994			38	8	
	France	1995			26	5	
	Germany	1997			28	11	
	Greece	1996			12	1	
	Hungary	1995			75	23	
	Italy	1993			30	4	
	Netherlands	1995			43	15	
	Poland	1996			43	8	
	Portugal	1996			38	7	
	Romania	1996			61	18	
	Spain	1995			45	6	
	Sweden	1996			22	12	
	United Kingdom	1997			48	31	
Siafakas *et al.*, 1995 [45]	Austria	1988-1991	NS		18	6	ICD 9 (490-493)
	Belgium				28	9	
	Bulgaria				11	6	
	Denmark				34	20	
	Finland				22	4	
	France				10	3	
	Greece				03	2	
	Hungary				40	16	
	Italy				25	8	
	Netherlands				20	5	
	Poland				29	8	
	Portugal				14	5	
	Romania				20	12	
	Spain				10	3	
	Sweden				11	5	
	United Kingdom				12	6	
Hospital population^e^							
Gudmundsson *et al.*, 2006^f ^[81]	SweNorFin^g^	2002	72.1 ± 8.7	293			ICD 10 (J40-J47)
Groeneweger *et al.*, 2003^h ^[79]	Netherlands	2001	70.6 ± 8.5	230			COPD-ATS
Almagro *et al.*, 2002^i ^[80]	Spain	1999	72.0 ± 9	220			COPD^j^

^a^Age was reported in various ways: mean age (±SD), or age range, min-max.^b^Not all European countries have COPD mortality data for 2007 in the Eurostat database. ^c^Total population (all ages). ^d^Greece does not have mortality data for asthma in 2007; these estimates correspond to J40-J47 and not to J40-J44, J47. ^e^Mortality rates in inpatients are of course not age-standardized but represent the number of deaths per 100,000 inhabitants. ^f^Mortality rates at 1 year for stages GOLD 1 and GOLD 2, GOLD 3, and GOLD 4 are 3.6, 6.9, and 13.7, respectively. ^g^This study concerned Sweden, Norway and Finland. ^h^Mortality rate at 6 months was 18. ^i^Mortality rates at 6 and 24 months were, respectively, 13.4 and 35.6. ^j^Clinical diagnosis of COPD and forced spirometry at discharge showing FEV_1 _<70% of the reference value and FEV_1_/FVC ratio <70%. Abbreviations used: ICD, International Classification of Diseases. COPD-ATS, chronic obstructive pulmonary disease as defined by the American Thoracic Society; NS, not specified; GOLD, Global Obstructive Lung Disease classification stage; FEV_1_, maximum expiratory volume in 1 second; FVC, forced vital capacity.

## Discussion

This study describes the variability of reported COPD epidemiological data in European countries. Although the search of documents was limited to the WOS and Eurostat databases, the collected information was sufficient to outline how international standardization of research methodology has evolved over time and has contributed to estimates of better quality. In that regard, whereas documents not included in the present study, such as unpublished information, might be available through national health surveys or other initiatives, their additional value to this study is likely to be low.

As shown, one of the main reasons for the variability of the reported epidemiological estimates is the use of different methods by different authors, hindering valid comparisons. For example, prevalence estimates were based on a wide variety of methods (for example, symptoms, physician reports, spirometry, models), different populations (for example, general population, hospital inpatients), different age distributions and different definitions of COPD. Studies based on a small number of patients and on interviews (symptoms or physician reports) are not as reliable as larger studies based on spirometry. Methods based on symptoms are neither specific nor sensitive for the diagnosis of COPD. The use of a screening questionnaire (and questionnaires may also vary from one study to another) does not allow thorough investigation of the specific characteristics of the patients, and the lack of functional investigations means that questions on progression from cough and sputum production to airflow obstruction remain unanswered [15]. The same applies to studies based on physician reports. Thus, in a study of a given general population sample, the estimated prevalence is, respectively, 5.6% and 26.1%, depending on whether one uses physician reports or spirometry [19]. The use of different severity scales is another important source of heterogeneity [19]. For example, among 212 patients included in the Obstructive Lung Disease in Northern Sweden surveys, 179 had COPD in the BTS classification, whereas all had COPD on the basis of the GOLD 2006 criteria, including patients with FEV_1 _≥80% of the predicted level (mild form) [49]. Because of these methodological problems and imprecise definitions, the terms *chronic bronchitis *and *chronic obstructive pulmonary disease *often were not differentiated in the past. However, these methodological limitations do not entirely explain the observed epidemiological variability. Other factors such as smoking and age probably contribute to the observed variability. A recent study considering COPD patients randomly selected from registers in a Swedish region indicated that only 59% had an initial diagnosis that involved spirometry [82], and in fact only longitudinal studies of young populations could allow early diagnosis and refine our knowledge of the natural history of COPD. In the recent largest international study on COPD, the BOLD study, prevalence data based on spirometry according to GOLD guidelines were collected at 12 sites in different countries, 5 of which were located in Europe [8]. Interestingly, these prevalence estimates are within the same range as those reported in the PLATINO study of five Latin American countries [9]. These results likely reflect the benefits of the international standardization of methodology. Nevertheless, the BOLD study reported significant differences across the different sites (*P *< 0.0001), but the authors underlined the danger of extrapolating data from such sites (a pool of about 150,000 people) to national populations. Therefore, the present national prevalence estimates that may be twice those from one European country to another (Table 1) should still be considered with caution.

As observed with regard to prevalence, a large epidemiological variability is observed with respect to COPD severity, but our study indicates that severity is the parameter for which recent standardization has resulted in the best improvement of estimates. Besides, severity is a very active research domain. In recent years, several composite scores, such as the BODE index (body mass index, airflow obstruction, dyspnea and exercise capacity), ADO index (age, dyspnea and airflow obstruction) or the DOSE index (dyspnea, airflow obstruction, smoking status and exacerbation frequency) [83,84], have been proposed for grading patients' disease severity. At present, the GOLD severity scale is the international standard, and our results indicate that recent estimates from the international studies based on this classification generally represent relatively low differences in the distribution of severity from one country to another within a given type of population.

This situation contrasts with the reported data on exacerbations for which the GOLD initiative did not recommend methodological guidelines. Unsurprisingly, exacerbation data clearly indicate that severe COPD is associated with more frequent and more severe exacerbations, but estimates of the frequency of exacerbations depend largely on the definition used [59,66,76]. Exacerbations are variously defined as a complex of respiratory symptoms or as a complex of respiratory symptoms requiring steroid or antibiotic treatment (Table 4). The influence of age and smoking status on exacerbations is poorly documented. The main COPD management objective is currently to prevent exacerbations. Most estimates concerning exacerbations come from therapeutic trials, which focus on selected populations and in which the frequency of exacerbations is used to judge treatment efficacy. As underlined by Aaron *et al.*[85], standardizing counts, analyses and method of reporting exacerbations are of major importance for a fair evaluation of treatments tested in randomized controlled trials.

Although several studies have reported COPD mortality data [45,78-81], the mortality data in the general population issued by Eurostat are those allowing the most standardized comparison between countries. The ICD is an international standard for categorizing mortality data, but the reliability of mortality estimates is undermined by problems of coding [86]. In addition, since some researchers think that there is a considerable overlap between asthma and COPD in adults, some COPD mortality estimates may be overestimated. Conversely, COPD mortality is also underreported [87], and causes of death remain to be standardized among patients with several comorbidities [16]. It may be difficult to get a great deal of consistency, because patients with COPD often do not die as a result of acute exacerbations of COPD or respiratory failure. More often they die as a result of cardiovascular causes, cancer, pneumonia and other related conditions. It can be challenging and difficult to decide which ICD code to use in recording the death certificate. More work is needed to come up with a realistic method of assessing the mortality rate in COPD. Finally, observed variations in COPD mortality rates across countries include variations in performance of the local healthcare systems with respect to COPD patient management, but indicators permitting such comparisons in specific settings are lacking.

The difficulties in comparing COPD studies outlined in this review can be attributed to two distinct reasons. First, the methodological bases may vary from one study to another, such as the definition of COPD. Nevertheless, our study also shows that efforts to establish congruence among COPD definitions and study methods are underway. Second, regardless of the methodology used for performing the study, the method of reporting results, such as exacerbation data, may vary from one report to another. This may also concern the reporting of important features related to the disease, such as smoking history. Smoking consumption (patients may be categorized in arbitrary classes of pack-year consumption) was used in some studies, whereas others used a three smoking status categorization (never smoker, former smoker, current smoker). There are current guidelines for reporting research, but they are based on the methodological type of the studies [88]. Our study strongly suggests that establishing reporting guidelines dedicated to COPD studies may constitute a major contribution to enhancing the value of COPD research and management by facilitating comparisons between studies.

## Conclusions

COPD is a major public health problem [89]. The wide range of prevalence, severity and mortality estimates across European countries cannot possibly correspond to real differences, but it remains difficult to assess the part of this heterogeneity due to methodological issues and the part of this heterogeneity due to countries' characteristics, such as differences in national smoking exposure. National prevalence estimates are still far from perfect, impairing fair comparisons from one country to another. The present study outlines that with time, congruence of COPD definitions, especially in terms of severity classification, and methodological standardization have resulted in more comparable epidemiological estimates, but much remains to be made in terms of exacerbation data. Many international efforts have been made to develop common rules for coding mortality data, but they concern all diseases, and potential improvements of COPD mortality data are likely to poorly depend on specific COPD programs. Efforts to establish common standardized COPD definitions and study methods are underway, and researchers are encouraged to do more to better assess the disease and the relative effectiveness of methods of patient management in different European Union member states. In that regard, the establishment of international guidelines for reporting research on COPD may constitute an important contribution. Indeed, the present review strongly supports the use of commonly agreed guidelines for bringing more uniformity to the findings reported in future studies. Our results emphasize the need for scientists and investigators to work harder at adopting common guidelines that may be imperfect but nevertheless acceptable, so that the epidemiological database can be richer, the burden of the disease can be better defined and the response of patients with COPD to treatment can be better evaluated.

## Competing interests

The authors declare that they have no competing interests.

## Authors' contributions

KA, CC and GH were responsible for the study's conception, design and data analysis as well as the drafting of the manuscript. KA also performed data collection.

## Pre-publication history

The pre-publication history for this paper can be accessed here:

http://www.biomedcentral.com/1741-7015/9/7/prepub

## Supplementary Material

Additional File 1 **Figure A1**. Chronic obstructive pulmonary disease (COPD) prevalence data. A graph of the prevalence estimates presented in Table 1. Click here for file

Additional File 2 **Figure A2**. COPD severity data. A graph of the severity data presented in Table 2. Click here for file

Additional File 3 **Figure A3**. COPD age-standardized mortality rates (number per 100,000). A graph of age-standardized mortality data presented in Table 5. Click here for file
