# Supplementary material for: Variability of the chronic obstructive pulmonary disease key epidemiological data in Europe: systematic review
Source: BMC Med. 2011 Jan 18;9:7. doi: 10.1186/1741-7015-9-7 (PMC3037331; doi:10.1186/1741-7015-9-7)
Supplement: Additional File 1 — Figure A1. Chronic obstructive pulmonary disease (COPD) prevalence data. A graph of the prevalence estimates presented in Table 1. [file 1741-7015-9-7-S1.pdf]

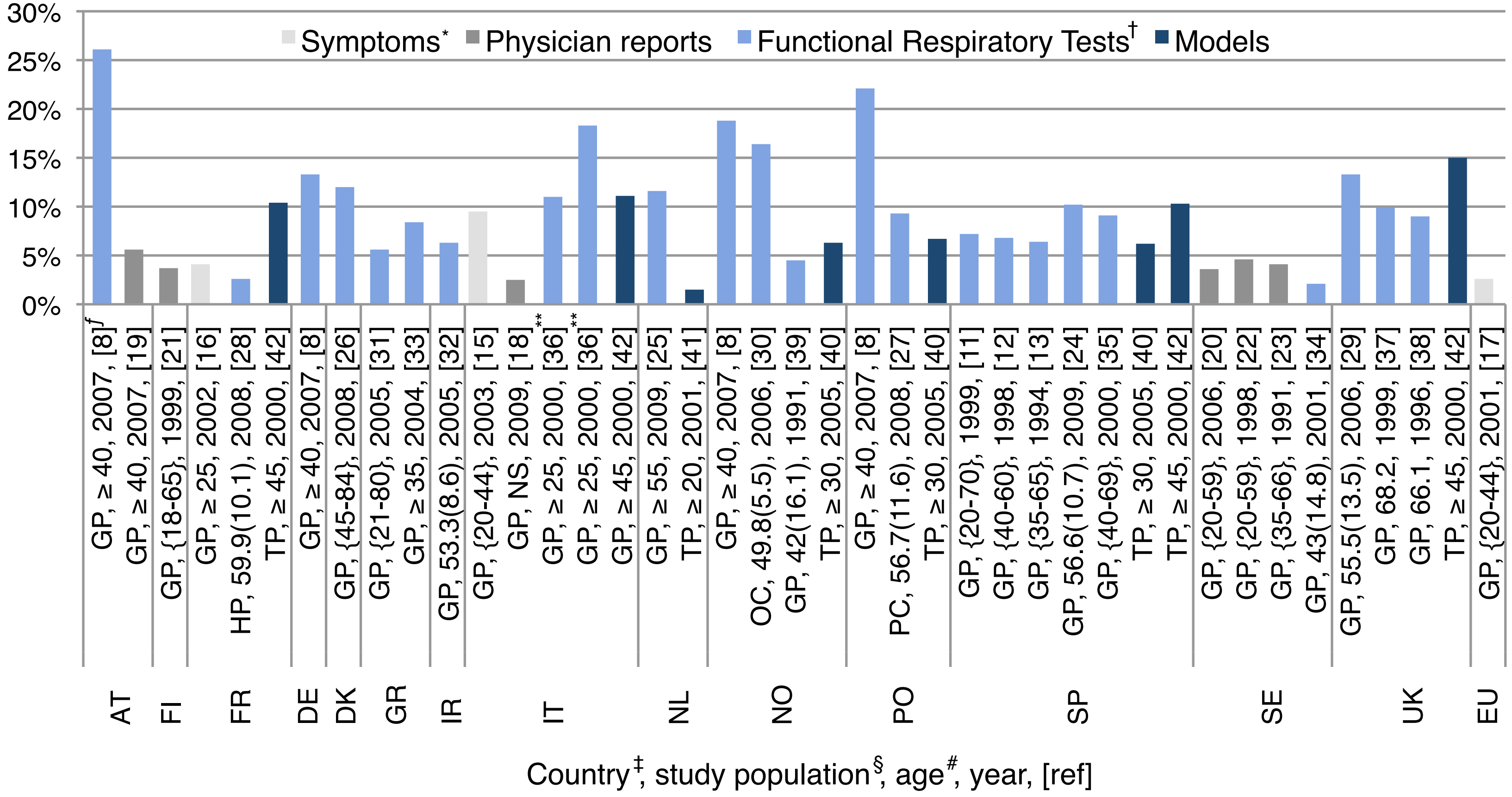

Figure A1 – COPD prevalence estimates (% of studied population).

Prevalence estimates are presented by country, type of population studied, age of studied population, year, and criterion (color of the bar) used for defining COPD patients.

Text abbreviations: COPD, Chronic Obstructive Pulmonary Disease; NS, Not specified.

*Symptoms correspond to cough and sputum at least 3 months each year. These patients had chronic bronchitis.

†Functional respiratory tests: FEV1/FVC<70% and FEV1<80% of predicted or FEV1/FVC<65% or FEV1/FVC<70% or FEV1/FVC< 88% (male)/ 89% (female).

‡Abbreviations for countries: AT, Austria; DK, Denmark; FI, Finland; FR, France; DE, Germany; GR, Greece; IR, Ireland; IT, Italy; NL, Netherlands; NO, Norway; PO, Poland; SP, Spain; SE, Sweden; UK, United Kingdom; EU, 16 European Union Countries.

§Abbreviations for types of population: GP, general population; HP, health prevention center; TP, total population; OC, occupational cohort; PC, primary care.

#Age was reported in various ways distinguished here as follows: "≥ x", minimum age; "x" or "x(y)", mean age or mean age (SD); "{x-y}", age range i.e. {min-max}.

ƒThese data are also reported in Schirnhofer, 2007 [19].

**Prevalence estimates based on the European Respiratory Society's statement or based on "clinical" criteria (FEV1/FVC < 70%) correspond to 11% and 18.3%, respectively.
