# Supplementary material for: Variability of the chronic obstructive pulmonary disease key epidemiological data in Europe: systematic review
Source: BMC Med. 2011 Jan 18;9:7. doi: 10.1186/1741-7015-9-7 (PMC3037331; doi:10.1186/1741-7015-9-7)
Supplement: Additional File 2 — Figure A2. COPD severity data. A graph of the severity data presented in Table 2. [file 1741-7015-9-7-S2.pdf]

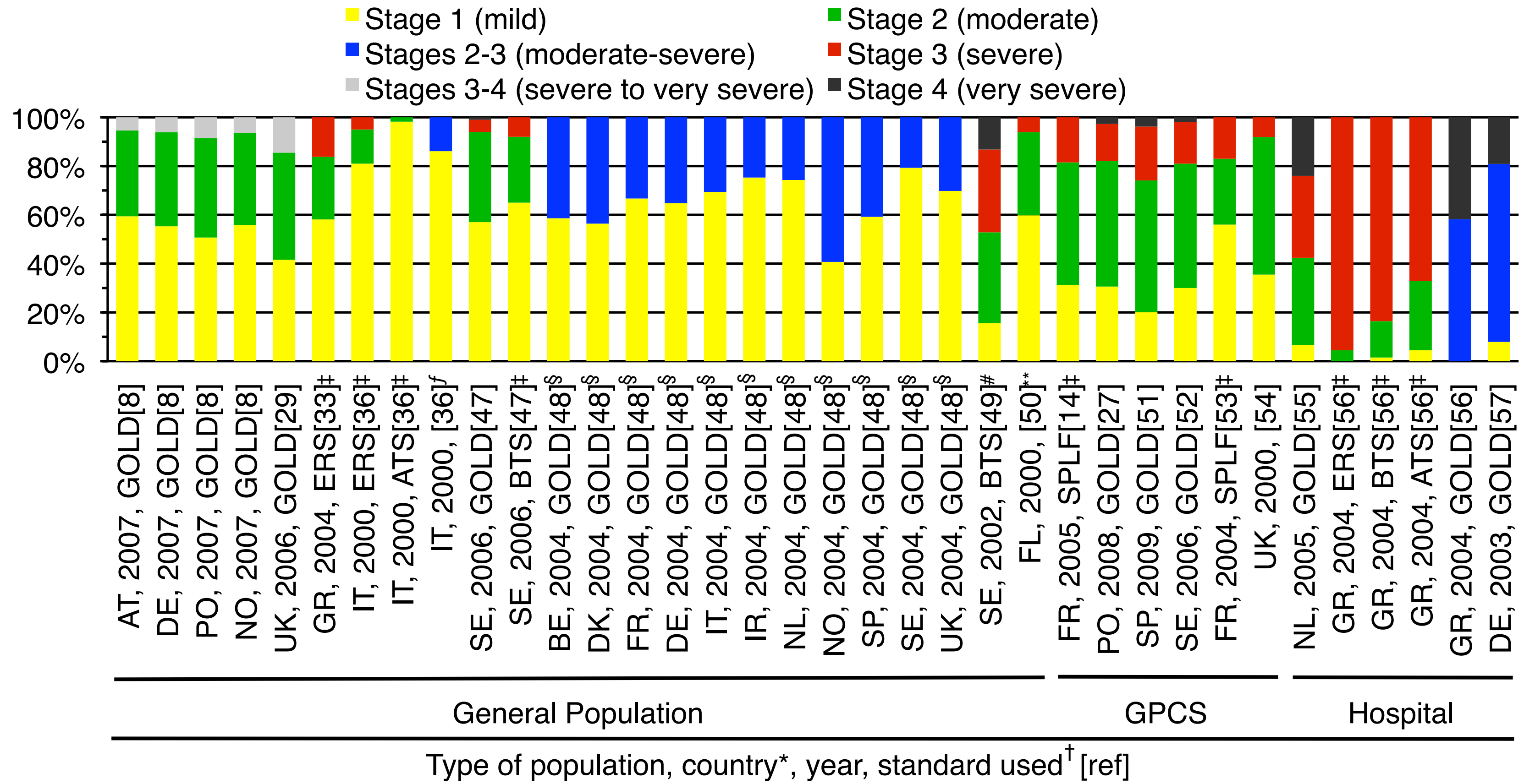

Figure A2 – COPD severity data.

Text abbreviations: COPD, Chronic Obstructive Pulmonary Disease; GPCS, General Practitioner and/or Chest Specialist.

*Abbreviations for countries: AT, Austria; DK, Denmark; FI, Finland; FR, France; DE, Germany; GR, Greece; IR, Ireland; IT, Italy; NL, Netherlands; NO, Norway; PO, Poland; SP, Spain; SE, Sweden; UK, United Kingdom; EU, 16 European Union Countries.

†Abbreviations for classifications: ERS, European Respiratory Society 1995 classification; ATS, American Thoracic Society 1995 classification; BTS, British Thoracic Society 1995 classification; SPLF, Société de Pneumonologie de Langue Française 1995 classification; GOLD, Global Obstructive Lung Disease 2001or 2003 or 2006 classifications.

‡The ERS, ATS, BTS, and SPLF 1995 classifications only define three stages.

§In this study, based on GOLD 2003 severity scale, the estimates in stage 0 defining persons at risk of COPD ranged from 51.7% in Switzerland to 89.8% in Spain.

#Persons with both FEV1/FVC<70% and FEV1≥80% (considered as having COPD in the GOLD 2006 classification but not in the BTS 1995 classification) were also included in the study.

ƒAirway obstruction of any degree was defined by FEV1/FVC<70% and the severity of obstruction was graded according to the FEV1 value: mild (FEV1≥70% of predicted) and moderate-severe (FEV1<70% of predicted)

**Airway obstruction of any degree was defined by FEV1/FVC < 80% and the severity of obstruction was graded according to the FEV1/FVC value: 70-79%, minimal to mild; 50-69%, moderate; <50%, severe.
