# Supplementary figures and images for: Variability of the chronic obstructive pulmonary disease key epidemiological data in Europe: systematic review
Source: BMC Med. 2011 Jan 18;9:7. doi: 10.1186/1741-7015-9-7 (PMC3037331; doi:10.1186/1741-7015-9-7)

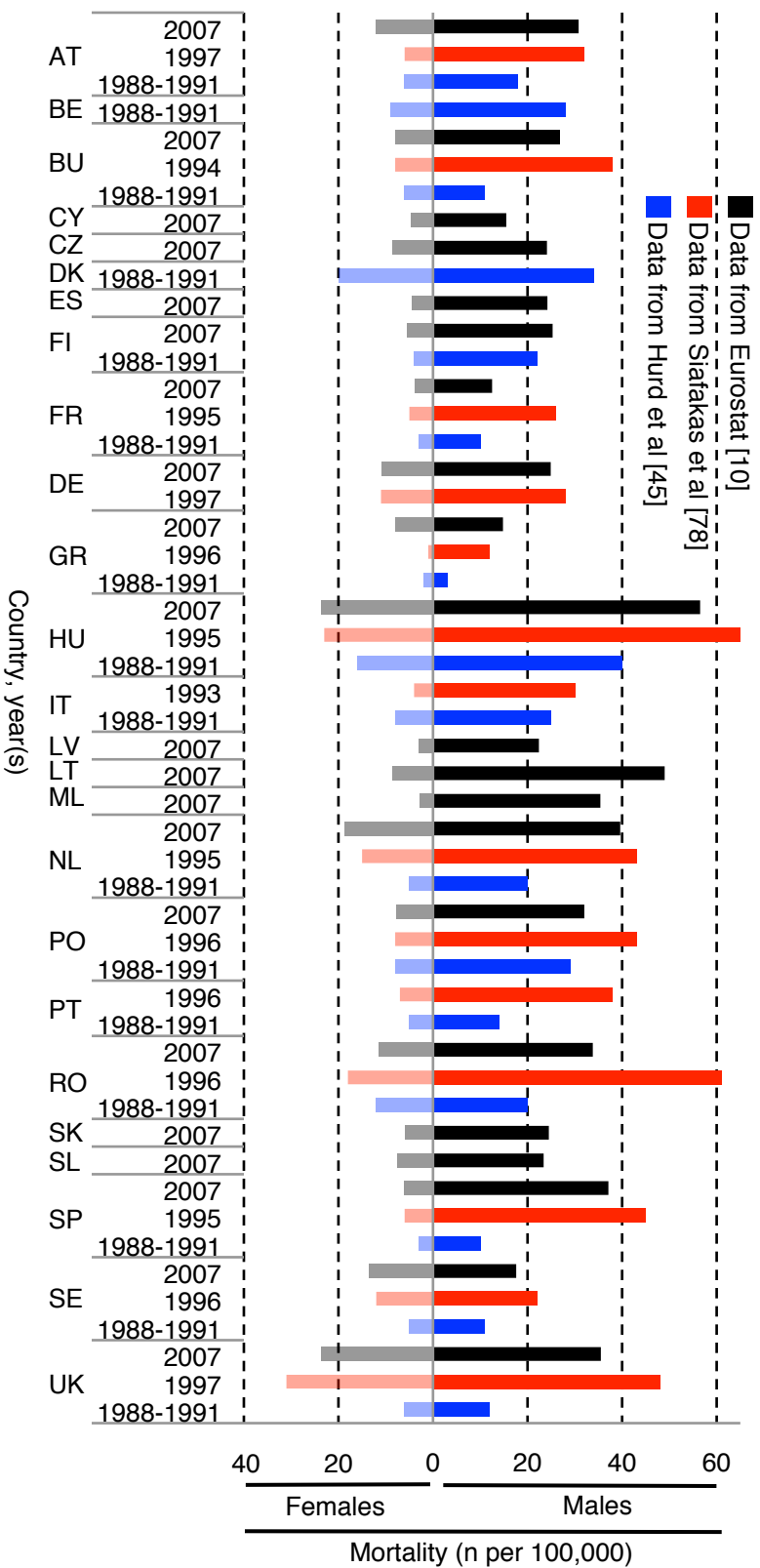

Supplement: Additional File 3 — Figure A3. COPD age-standardized mortality rates (number per 100,000). A graph of age-standardized mortality data presented in Table 5. [file 1741-7015-9-7-S3.pdf]
